# Geographic and Racial Disparities in Maternal and Infant Mortality Rates Across U.S. States: A Population-Based Analysis

**DOI:** 10.7759/cureus.108738

**Published:** 2026-05-12

**Authors:** Erin I Duffy, Diamond Posley, Irene Li, George Grant, Gul Aman

**Affiliations:** 1 Emergency Medicine, Stony Brook University, Stony Brook, USA; 2 Health Administration, Stony Brook University, Stony Brook, USA

**Keywords:** health disparities, health equity, infant mortality, maternal mortality, perinatal outcomes, population health, racial disparities, social determinants of health, united states

## Abstract

Background

Maternal and infant mortality remain critical indicators of population health in the United States, with persistent geographic and racial/ethnic disparities. Although these outcomes are often examined separately, less attention has been given to whether jurisdictions with higher infant mortality also experience higher maternal mortality. This ecological, jurisdiction-level study evaluated recent patterns in maternal and infant mortality across the 50 U.S. states and the District of Columbia, examined geographic and racial/ethnic disparities, and assessed the association between infant mortality rate (IMR) and maternal mortality rate (MMR).

Methods

This population-based analysis used publicly available Centers for Disease Control and Prevention (CDC) WONDER data. The primary jurisdiction-level analysis used pooled 2020-2024 infant and maternal mortality data to assess overall geographic patterns and the association between IMR and MMR. Secondary race/ethnicity-specific analyses used pooled 2020-2023 data because CDC Linked Birth/Infant Death Records were available through 2023. Race/ethnicity categories included non-Hispanic White, non-Hispanic Black, Hispanic, non-Hispanic Asian, non-Hispanic Native Hawaiian or Other Pacific Islander (NHOPI), and non-Hispanic American Indian and Alaska Native (AIAN). IMR was calculated per 1,000 live births, and MMR per 100,000 live births. Pearson correlation and analysis of variance (ANOVA) were used.

Results

Using pooled 2020-2024 data, overall IMR ranged from 3.43 to 9.04 deaths per 1,000 live births, with the lowest rates in Massachusetts, New Jersey, and New Hampshire and the highest in Mississippi, Arkansas, and Louisiana. Overall, MMR ranged from 0.00 to 41.76 deaths per 100,000 live births; zero and low-count values were interpreted cautiously because maternal mortality is rare and jurisdiction-level estimates may be unstable. The lowest reported MMRs were in Vermont, California, and Utah, and the highest were in Tennessee, Louisiana, and Mississippi. The South had the highest mean IMR and MMR, while the Northeast had the lowest. In secondary 2020-2023 race/ethnicity-specific analyses, disparities persisted across expanded racial/ethnic categories, although jurisdiction-level estimates for smaller groups were limited by low counts and data suppression. Black populations had higher IMR and MMR than White and Hispanic populations. AIAN and NHOPI populations also had elevated aggregate infant mortality, and AIAN populations had elevated aggregate maternal mortality. Male infants had higher mortality than female infants. Overall, IMR and MMR were positively correlated across jurisdictions.

Conclusions

Geographic and racial/ethnic disparities in maternal and infant mortality remain substantial in recent U.S. data, with the greatest burden concentrated in Southern jurisdictions and among Black populations. Elevated rates were also observed among AIAN and NHOPI populations, where data were available. The jurisdiction-level association between IMR and MMR suggests that these outcomes may share overlapping population-level determinants, although causal or individual-level inferences cannot be made from this ecological analysis. Future studies should examine structural, socioeconomic, and healthcare-system factors that may explain the parallel distribution of maternal and infant mortality across jurisdictions.

## Introduction

Despite advances in medical care, maternal and infant mortality remain persistent public health concerns in the United States, with disparities across geographic regions and racial and ethnic groups [1,2]. Prior research has demonstrated that Southeastern states consistently exhibit the highest mortality rates, while the Northeast and West tend to have lower rates [1,3-5]. Additionally, Black populations experience maternal mortality rate (MMR) and infant mortality rate (IMR) two to three times higher than White populations, highlighting longstanding inequities in health outcomes [1,2].

These disparities are increasingly understood to reflect structural factors, including differences in healthcare access, quality of care, and broader social determinants of health (SDOH), rather than biological differences alone [4-6]. Factors such as poverty, education, housing, environmental exposures, insurance coverage, and healthcare infrastructure influence both maternal health and infant outcomes across the perinatal period. Prior evidence suggests that racial and geographic disparities have remained persistent over time, even as some overall mortality rates have improved, with relative gaps between Black and White populations showing little meaningful narrowing [2,7,8]. For example, IMRs have declined over recent decades, yet racial gaps have remained substantial [7], while maternal mortality disparities have remained pronounced and, in some analyses, have widened in recent years [7,8].

Although Black-White disparities have been widely documented, other racial and ethnic groups also experience important and sometimes underrecognized disparities. American Indian and Alaska Native (AIAN) and Native Hawaiian or Other Pacific Islander (NHOPI) populations have elevated IMRs compared with White and Asian populations, while AIAN populations have also experienced high and increasing maternal mortality in prior state-level analyses [1,2,9-11]. Asian populations often have lower aggregate IMRs in national data, although broad racial categories may mask subgroup heterogeneity [2]. Similarly, NHOPI outcomes may be obscured when combined with Asian populations, and AIAN mortality may be underestimated because of racial misclassification in death certificate data [10,11]. These issues highlight the importance of examining expanded racial and ethnic categories where data are available, while also recognizing that small population sizes, data suppression, and unstable state-level estimates limit interpretation.

Contemporary analysis is also important because recent changes in healthcare access, maternity care availability, policy interventions, and the disproportionate impact of the COVID-19 pandemic may have influenced the magnitude and distribution of maternal and infant mortality disparities [7]. Because maternal and infant mortality are distinct outcomes that occur across the same perinatal continuum, examining whether they demonstrate similar jurisdiction-level patterning may provide insight into shared population-level determinants. However, such associations must be interpreted ecologically and should not be assumed to reflect individual-level or causal relationships.

This study uses recent national data to evaluate current geographic and racial/ethnic patterns in maternal and infant mortality across the 50 U.S. states and the District of Columbia. The primary analysis examines overall jurisdiction-level infant and maternal mortality using pooled 2020-2024 data to improve temporal comparability and estimate stability. Secondary analyses examine race/ethnicity-specific patterns using pooled 2020-2023 data, including non-Hispanic White, non-Hispanic Black, Hispanic, non-Hispanic Asian, non-Hispanic NHOPI, and non-Hispanic AIAN populations. We also assess whether jurisdiction-level IMR and MMR are correlated, with the goal of evaluating whether these outcomes demonstrate parallel population-level patterning in contemporary data.

## Materials and methods

This study utilized publicly available secondary data obtained from the Centers for Disease Control and Prevention (CDC) WONDER database [12-14]. CDC WONDER queries were performed on May 3, 2026. Detailed query parameters are provided in Supplemental Appendix 1.

The primary analysis examined overall jurisdiction-level IMRs and MMRs pooled from 2020 through 2024. Data were pooled to improve the stability of the estimate and temporal comparability. For this analysis, infant deaths were obtained from CDC WONDER Underlying Cause of Death files by selecting deaths occurring before age one year. Maternal deaths were obtained from CDC WONDER Underlying Cause of Death files using ICD-10 codes A34, O00-O95, and O98-O99. Live birth denominators were obtained from CDC WONDER Natality data for the same years and jurisdictions.

Secondary race/ethnicity-specific analyses were conducted using pooled 2020 through 2023 data to allow use of the CDC Linked Birth/Infant Death Records, the preferred source for race/ethnicity-specific infant mortality estimates, and available through 2023 at the time of analysis. For these secondary analyses, infant deaths were obtained from CDC Linked Birth/Infant Death Records and grouped by jurisdiction and maternal race/ethnicity. Maternal deaths were obtained from CDC WONDER Underlying Cause of Death files using the same ICD-10 codes as previously mentioned. Live birth denominators were obtained from CDC WONDER Natality data. Race/ethnicity categories included non-Hispanic White, non-Hispanic Black, Hispanic, non-Hispanic Asian, non-Hispanic NHOPI, and non-Hispanic AIAN. Hereafter, White, Black, Asian, NHOPI, and AIAN refer to non-Hispanic populations. Analyses included the 50 U.S. states and the District of Columbia (n = 51).

Infant mortality was defined as death occurring within the first year of life. IMR was calculated as the number of infant deaths divided by the number of live births, multiplied by 1,000. Maternal mortality was defined using ICD-10 codes related to pregnancy, childbirth, and the postpartum period (A34, O00-O95, O98-O99), capturing deaths occurring during pregnancy or within 42 days postpartum. MMR was calculated as the number of maternal deaths divided by the number of live births, multiplied by 100,000.

CDC-suppressed cells were coded as missing and excluded from analyses that required that value. Values reported as 0.00 were treated differently depending on the analysis. In the primary, overall jurisdiction-level analysis, 0.00 values were retained because the analysis used pooled 2020-2024 data, denominators were larger, and only one jurisdiction had a reported MMR value of 0. In race/ethnicity-specific jurisdiction-level analyses, 0.00 values were retained for descriptive display but excluded from state-level mean calculations and inferential analyses. This was done because zero values in these analyses generally occurred in smaller racial/ethnic strata, where a rate of 0.00 may be unstable for between-state comparisons. For this reason, aggregate pooled race/ethnicity-specific rates were emphasized when comparing smaller racial/ethnic groups.

Given the ecological nature of the dataset, all results are presented as population-level associations. Analyses were conducted at the jurisdiction level and should not be interpreted as individual-level or causal comparisons. For infant mortality, regional differences in overall 2020-2024 IMR were evaluated using one-way analysis of variance (ANOVA) across U.S. Census regions. Race/ethnicity-specific IMR differences were evaluated using one-way ANOVA with available 2020-2023 jurisdiction-level data. Differences between male and female IMR were evaluated using paired t-tests across jurisdictions.

For maternal mortality, regional differences in overall 2020-2024 MMR were evaluated using one-way ANOVA across U.S. Census regions. Race/ethnicity-specific MMR comparisons were limited to White, Black, and Hispanic populations for inferential analysis because jurisdiction-level estimates for Asian, NHOPI, and AIAN populations were sparse or frequently suppressed. Aggregate pooled MMR estimates for Asian, NHOPI, and AIAN populations were reported descriptively.

The primary IMR/MMR comparison used overall jurisdiction-level rates pooled from 2020 through 2024. The association between overall jurisdiction-level IMR and MMR was assessed using Pearson correlation analysis. This approach was intended to describe the unadjusted population-level association between IMR and MMR, rather than to identify independent predictors. Spearman’s rank correlation was also calculated as a robustness check to determine whether the association remained similar when using a rank-based correlation method.

Before analysis, distributions and variance patterns were reviewed descriptively. Because sample sizes, data availability, and variability differed across regional and racial/ethnic groups, ANOVA results were interpreted cautiously alongside descriptive 95% confidence intervals, particularly when cell suppression or low counts limited estimate stability. Ninety-five percent confidence intervals were feasible. Very small p-values were reported as p < 0.001. A p-value of <0.05 was considered statistically significant.

Choropleth maps were created using Datawrapper (Datawrapper GmbH, Berlin, Germany), and Microsoft Excel (Microsoft Corp., Redmond, WA, USA) was used for additional data visualization.

## Results

Infant mortality 

**Figure 1:**
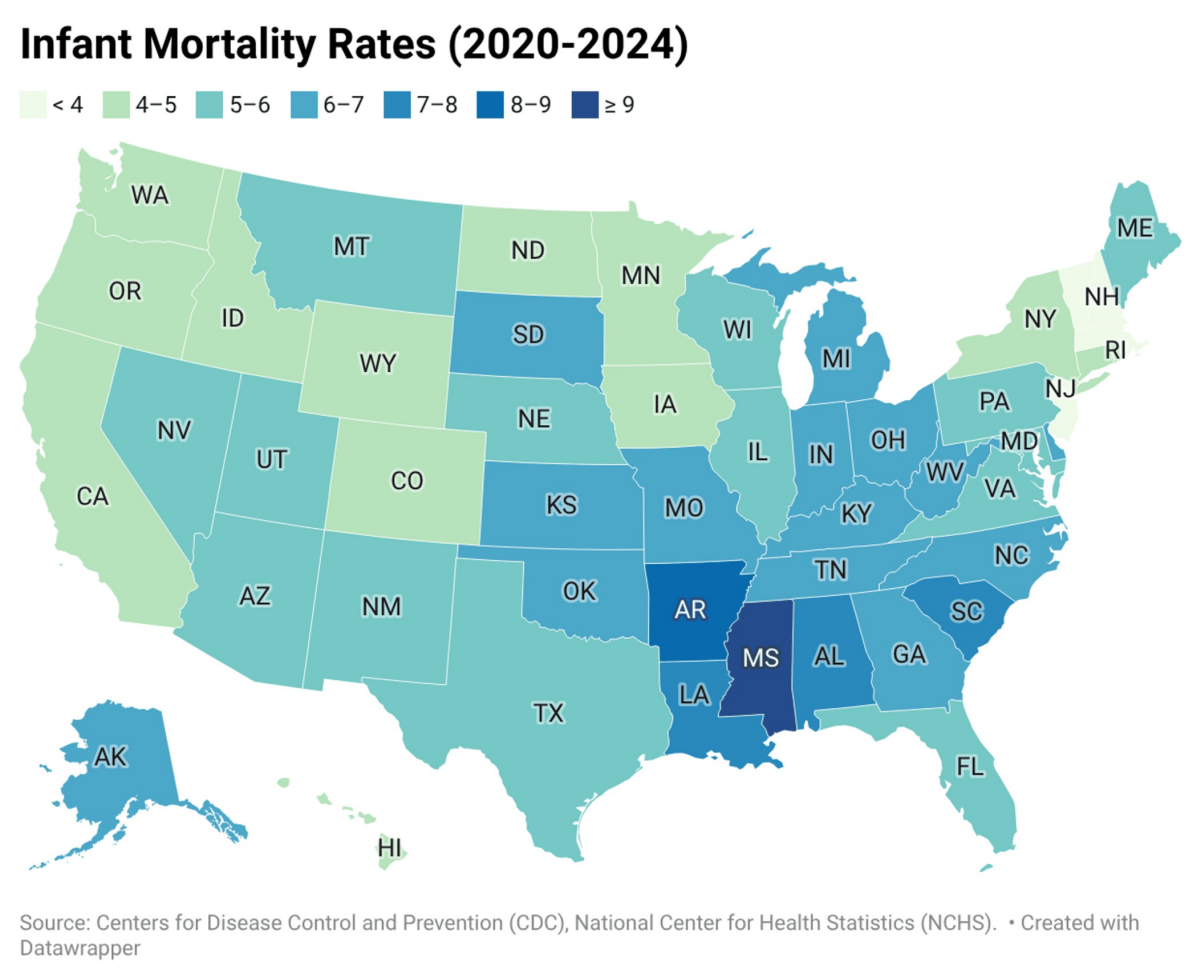
Variations in infant mortality rates by region of the United States Source: Centers for Disease Control and Prevention (CDC), National Center for Health Statistics (NCHS); image created using the Datawrapper platform (Datawrapper GmbH, Berlin, Germany).

IMRs from pooled 2020-2024 varied substantially across jurisdictions (n = 51), ranging from 3.43 to 9.04 deaths per 1,000 live births. The lowest rates were observed in Massachusetts (3.43), New Jersey (3.61), and New Hampshire (3.64), while the highest occurred in Mississippi (9.04), Arkansas (8.10), and Louisiana (7.38) (Figure 1). Regional variation was pronounced. Mean IMR was 4.30 ± 0.81 in the Northeast (n = 9), 5.86 ± 0.86 in the Midwest (n = 12), 6.68 ± 0.91 in the South (n = 17), and 5.03 ± 0.64 in the West (n = 13). These differences were statistically significant (one-way ANOVA, F(3, 47) = 19.64, p < 0.001) (Table 1). 

**Table 1:** Variation in infant mortality rates by region IMR: Infant Mortality Rate; SD: Standard Deviation; 95% CI: 95% Confidence Interval

Region	n	Mean IMR	SD	95% CI
Northeast	9	4.30	0.81	3.68-4.92
Midwest	12	5.86	0.86	5.31-6.41
South	17	6.68	0.91	6.21-7.15
West	13	5.03	0.64	4.64-5.42

**Figure 2:**
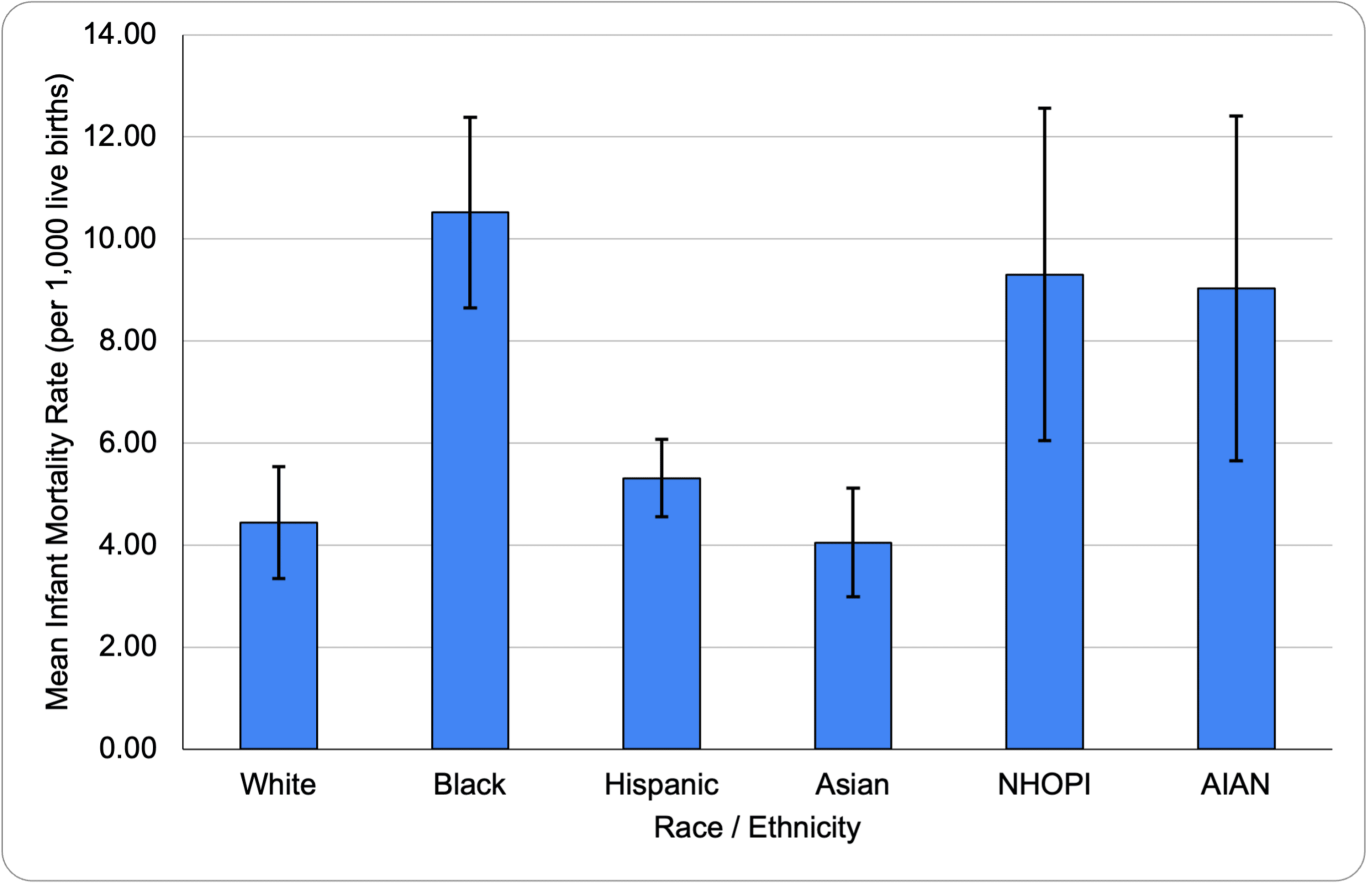
Mean infant mortality rate at jurisdiction level by race/ethnicity (2020-2023) Error bars represent standard deviation (SD). NHOPI: Native Hawaiian or Other Pacific Islander; AIAN: American Indian and Alaska Native

**Figure 3:**
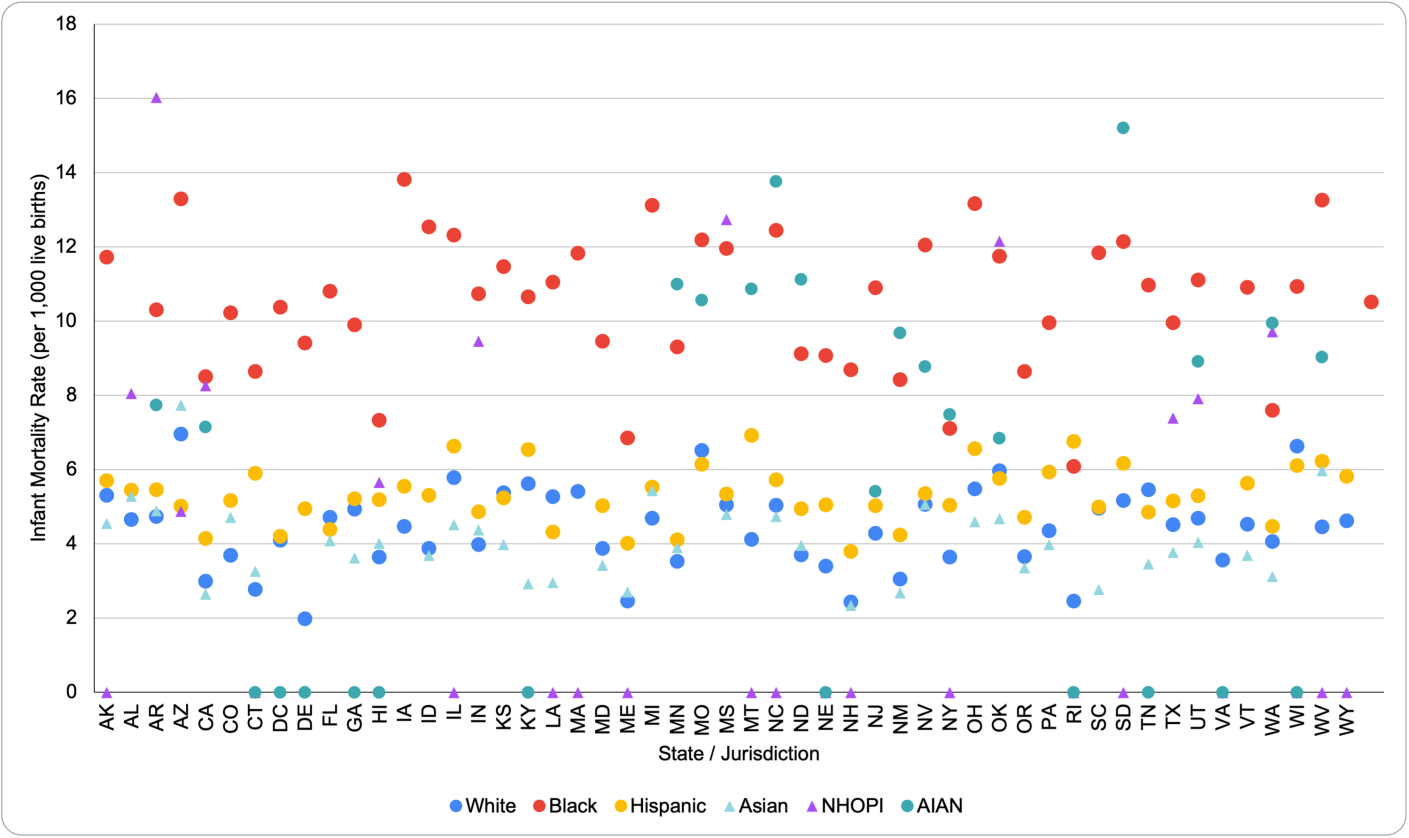
Infant mortality rate by race/ethnicity and jurisdiction (2020-2023) NHOPI: Native Hawaiian or Other Pacific Islander; AIAN: American Indian and Alaska Native

Racial disparities observed in the 2020-2023 data set were one of the most prominent findings. Aggregate national IMR estimates, which include data suppressed at the state level, were 4.44 among White infants, 10.68 among Black infants, 4.85 among Hispanic infants, 3.44 among Asian infants, 7.90 among NHOPI infants, and 8.33 among AIAN infants. When looking at jurisdiction-level IMR, mean IMR was 4.43 ± 1.10 among White infants (n = 51), 10.52 ± 1.87 among Black infants (n = 47), 5.31 ± 0.76 among Hispanic infants (n = 49), 4.05 ± 1.07 among Asian infants (n = 37), 9.30 ± 3.26 for NHOPI infants (n = 11), and 9.03 ± 3.38 for AIAN infants (n = 17), with statistically significant differences across groups (one-way ANOVA, F(5, 206) = 104.13, p < 0.001) (Table 2 and Figure 2). Across nearly all jurisdictions with available data, Black IMRs were consistently higher than those among White, Asian, and Hispanic populations (Figure 3). The exception was Rhode Island, where Black infants had lower mortality rates than Hispanic infants. AIAN and NHOPI IMRs were also higher than White, Asian, and Hispanic rates in jurisdictions with available data. 

**Table 2:** Jurisdiction-level infant mortality rates by race/ethnicity (2020-2023) IMR: Infant Mortality Rate; SD: Standard Deviation; 95% CI: 95% Confidence Interval; NHOPI: Native Hawaiian or Other Pacific Islander; AIAN: American Indian and Alaska Native

Race/Ethnicity	n	Mean IMR	SD	95% CI
White populations	51	4.43	1.10	4.12-4.74
Black populations	47	10.52	1.87	9.97-11.07
Hispanic populations	49	5.31	0.76	5.09-5.53
Asian populations	37	4.05	1.07	3.69-4.41
NHOPI populations	11	9.30	3.26	7.11-11.49
AIAN populations	17	9.03	3.38	7.29-10.77

Figure 3 shows that Black IMRs were higher than White, Hispanic, and Asian IMRs across most jurisdictions with available data. AIAN and NHOPI IMRs were also elevated in several jurisdictions, although interpretation was limited by the smaller number of jurisdictions with available non-suppressed data. AIAN infant mortality was highest in South Dakota, and NHOPI infant mortality was highest in Arizona among jurisdictions with available data. Several jurisdictions reported AIAN or NHOPI IMRs of 0.00; these values were retained for graphical display but excluded from state-level mean calculations and inferential analyses because they occurred in small population strata, where rates are unstable for between-state comparisons.

Looking at the 2020-2024 data, male infants demonstrated higher mortality rates than female infants across jurisdictions with available data (n = 51). Mean IMR across states was 6.08 ± 1.30 among male infants and 5.19 ± 1.13 among female infants, corresponding to a mean difference of 0.89 deaths per 1,000 live births (paired t-test, t = 12.83; 95% CI 0.75-1.03; p < 0.001).

Maternal mortality 

**Figure 4:**
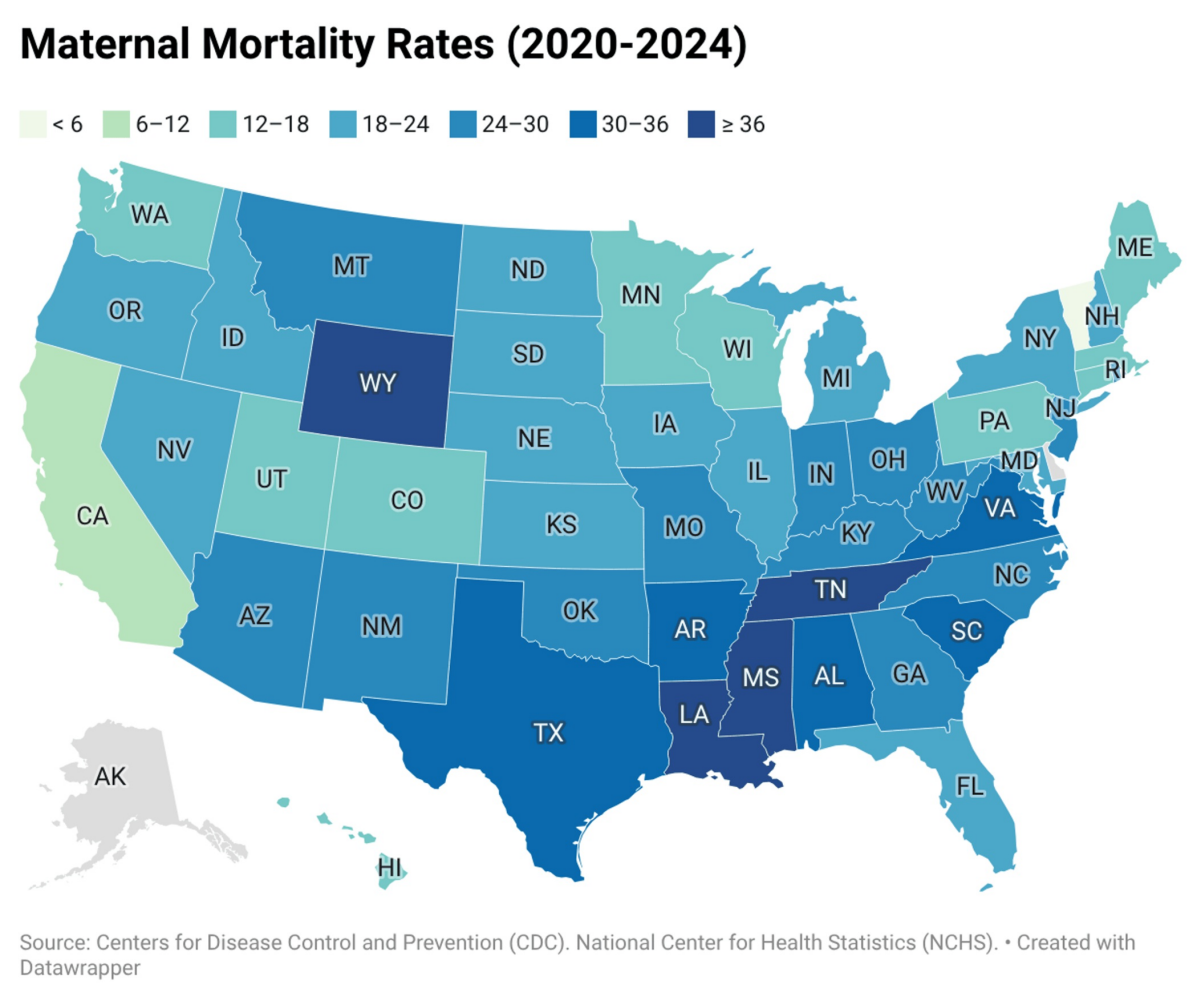
Variations in maternal mortality rates by region of the United States Source: Centers for Disease Control and Prevention (CDC), National Center for Health Statistics (NCHS); image created using the Datawrapper platform (Datawrapper GmbH, Berlin, Germany).

MMRs from the pooled 2020-2024 dataset also demonstrated wide variation across jurisdictions (n = 51), ranging from 0.00 to 41.76 deaths per 100,000 live births (Figure 4). The lowest reported rates were observed in Vermont (0.00), California (10.81), and Utah (12.18). Values reported as 0.00 in the source dataset were retained as reported and reflect jurisdictions in which no deaths matching the query criteria were recorded during the study period. The highest occurred in Tennessee (41.76), Louisiana (39.36), and Mississippi (37.52). Regional differences in MMR were also present. Mean MMR was 17.47 ± 7.70 in the Northeast (n = 9), 21.88 ± 4.06 in the Midwest (n = 12), 30.01 ± 5.83 in the South (n = 16), and 21.17 ± 7.85 in the West (n = 12). These differences were statistically significant across regions (one-way ANOVA, F(3, 45) = 8.97, p < 0.001). Regional means, standard deviations, and 95% confidence intervals are shown in Table 3.

**Table 3:** Variation in maternal mortality rates by region MMR: Maternal Mortality Rate; SD: Standard Deviation; 95% CI: 95% Confidence Interval

Region	n	Mean MMR	SD	95% CI
Northeast	9	17.47	7.70	11.55-23.39
Midwest	12	21.88	4.06	19.30-24.46
South	16	30.01	5.83	26.90-33.12
West	12	21.17	7.85	16.18-26.16

**Figure 5:**
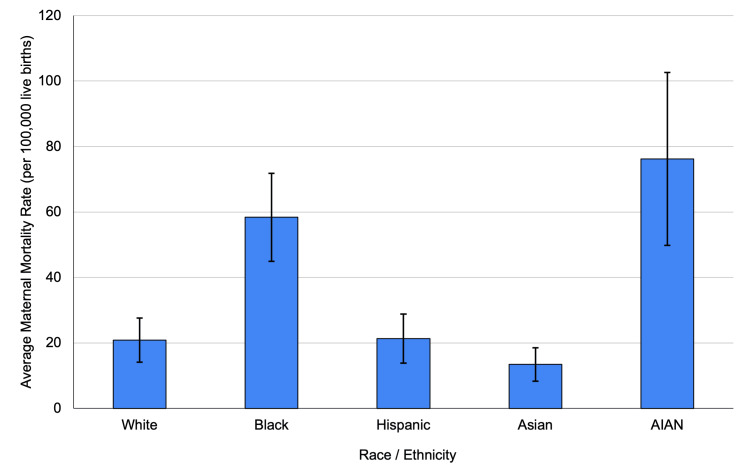
Mean maternal mortality rate at jurisdiction level by race/ethnicity (2020-2023) Error bars represent standard deviation (SD). AIAN: American Indian and Alaska Native

**Figure 6:**
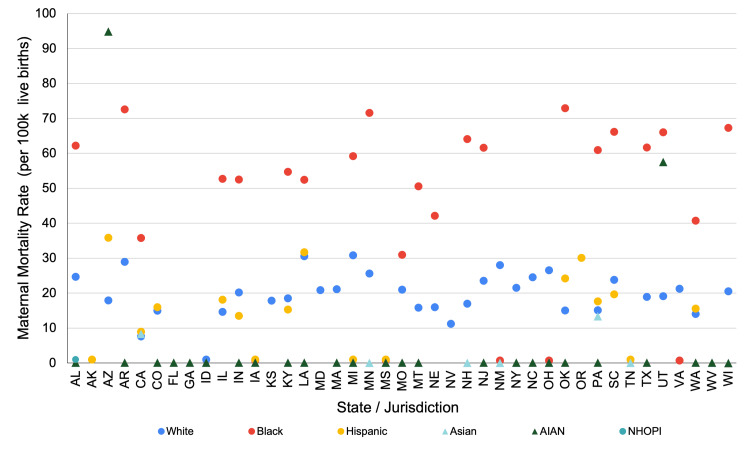
Maternal mortality rate by race/ethnicity and jurisdiction (2020-2023) NHOPI: Native Hawaiian or Other Pacific Islander; AIAN: American Indian and Alaska Native

Racial disparities were also present in the maternal mortality data. Aggregate pooled MMR estimates for 2020-2023, which include data suppressed at the state level, were 19.89 for White women, 56.32 for Black women, 18.73 for Hispanic women, 13.25 for Asian women, 38.08 for NHOPI women, and 63.94 for AIAN women. In the jurisdiction-level inferential analysis of White, Black, and Hispanic populations, mean state-level MMR was 20.90 ± 6.75 among White populations (n = 39), 58.40 ± 13.45 among Black populations (n = 25), and 21.36 ± 7.49 among Hispanic populations (n = 16), with statistically significant differences across groups (one-way ANOVA, F(2, 77) = 132.02, p < 0.001) (Table 4 and Figure 5). Across jurisdictions with available non-zero rates, Black MMRs were consistently higher than White and Hispanic MMRs (Figure 6). Aggregate pooled rates also suggested elevated MMR among AIAN and NHOPI populations; however, jurisdiction-level estimates for Asian and AIAN populations were sparse, and jurisdiction-level NHOPI MMR could not be calculated because state-level values were suppressed or reported as 0.00 across jurisdictions.

**Table 4:** Jurisdiction-level maternal mortality rates by race/ethnicity (2020-2023) *The AIAN jurisdiction-level MMR estimate is based on two jurisdictions with available non-zero, non-suppressed data and should be interpreted with caution. NHOPI is not shown because the jurisdiction-level mean MMR could not be calculated due to suppressed or 0.00 values across jurisdictions. MMR: Maternal Mortality Rate; SD: Standard Deviation; 95% CI: 95% Confidence Interval; NHOPI: Native Hawaiian or Other Pacific Islander; AIAN: American Indian and Alaska Native

Race/Ethnicity	n	Mean MMR	SD	95% CI
White populations	39	20.90	6.75	18.71-23.09
Black populations	25	58.40	13.45	52.85-63.95
Hispanic populations	16	21.36	7.49	17.37-25.35
Asian populations	3	13.43	5.08	0.81-26.05
AIAN populations	2	76.23	26.44	0.00-313.78*

Figure 6 demonstrates that among jurisdictions with available data, Black MMR was higher than White and Hispanic MMR across most jurisdictions. AIAN MMR was elevated in Arizona and Utah, although jurisdiction-level estimates for AIAN and NHOPI populations were limited by 0.00 values and CDC suppression in many states. These findings highlight the instability of state-level maternal mortality estimates for smaller population groups when rates are based on rare events and small denominators. Zero values were retained for graphical display but excluded from state-level mean calculations and inferential analyses for race/ethnicity-specific comparisons.

Correlation between infant and maternal mortality

**Figure 7:**
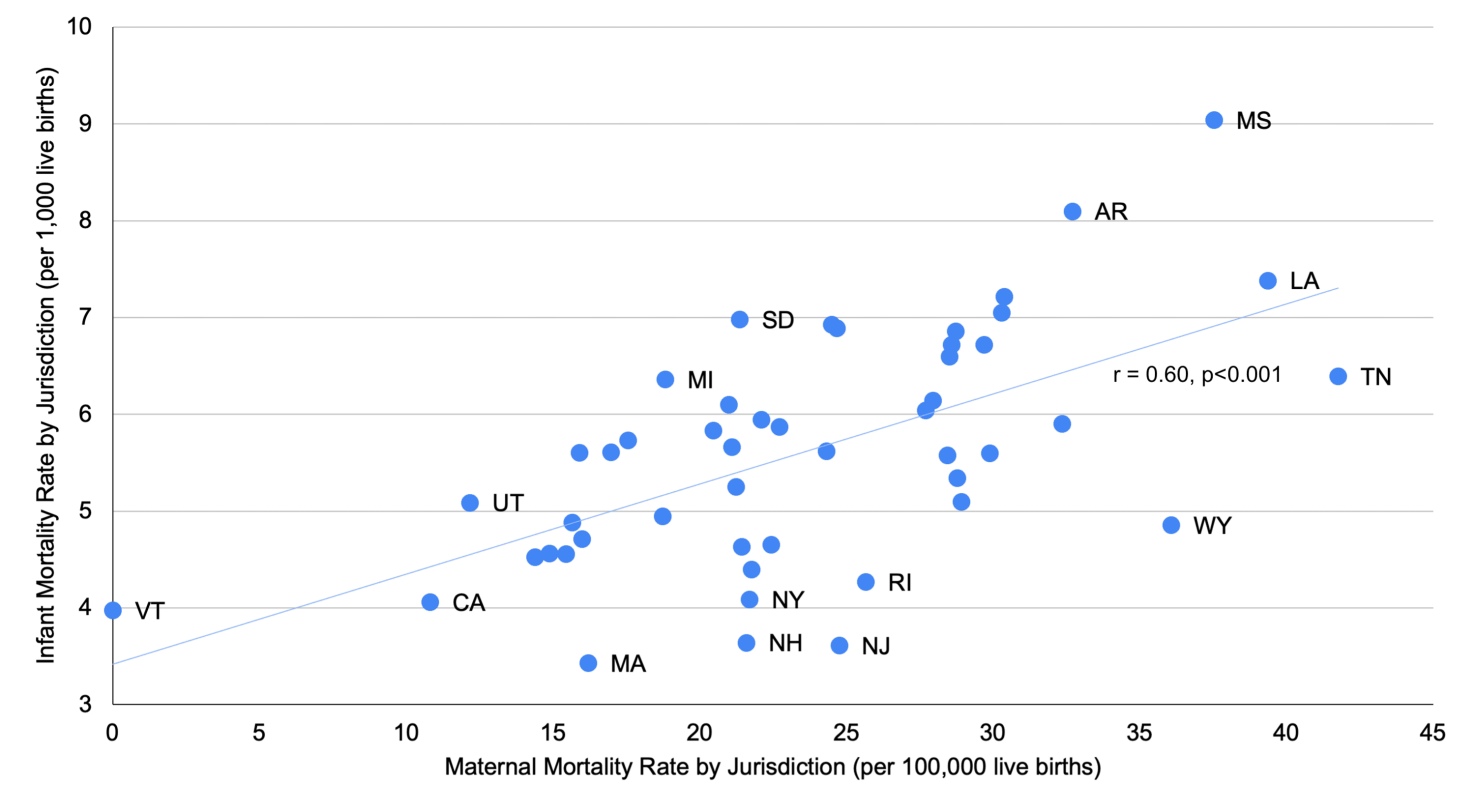
Association between maternal and infant mortality rates by jurisdiction (2020-2024)

There was a statistically significant positive correlation between IMR and MMR across jurisdictions (n = 49), indicating that jurisdictions with higher infant mortality also tended to have higher maternal mortality (Pearson correlation, r = 0.60, 95% CI 0.39-0.76, p < 0.001) (Figure 7). The association remained similar using Spearman rank correlation (ρ = 0.58, 95% CI 0.35-0.74, p < 0.001), supporting the robustness of the association to non-normality and visually discordant values.

Although most jurisdictions followed the overall positive trend, several states deviated visually from the fitted line. Wyoming, New Jersey, New Hampshire, and Massachusetts had lower IMRs than would be expected based on their MMRs alone. In contrast, Arkansas and Mississippi had high IMRs in the setting of elevated MMRs. Louisiana was among the jurisdictions with the highest values for both measures, while Vermont had the lowest maternal mortality value in this analysis and a relatively low IMR.

## Discussion

This ecological analysis of recent national data demonstrates that substantial geographic and racial/ethnic disparities in both infant and maternal mortality persist across the United States and the District of Columbia. In the primary jurisdiction-level analysis using pooled 2020-2024 data, both IMR and MMR were highest in the South and lowest in the Northeast, with statistically significant regional differences for both outcomes. The similar geographic distribution of these outcomes is notable because maternal and infant mortality are distinct clinical endpoints, yet both were elevated in many of the same jurisdictions. This supports the interpretation that these outcomes may share upstream population-level determinants, although the ecological study design does not allow causal or individual-level inference.

Racial and ethnic disparities were also prominent. In secondary analyses using pooled 2020-2023 data, Black populations had substantially higher IMR and MMR than White and Hispanic populations. These findings are consistent with prior literature demonstrating persistent Black-White disparities in maternal and infant outcomes [1,2,7,8]. Importantly, the expanded race/ethnicity-specific analysis also demonstrated elevated infant mortality among AIAN and NHOPI populations and elevated maternal mortality among AIAN populations when data were available. These findings add value beyond confirming known Black-White disparities by highlighting the importance of including smaller racial and ethnic groups in contemporary surveillance [1,9-11]. However, jurisdiction-level estimates for NHOPI, AIAN, and Asian populations were frequently limited by small numerators, zero values, and CDC suppression. Therefore, these estimates should be interpreted cautiously and viewed as descriptive rather than definitive state-level comparisons.

Hispanic populations had lower infant mortality than Black populations in this analysis, which is consistent with the Hispanic or Latino epidemiologic paradox described in prior literature [15]. This paradox refers to the observation that some Hispanic populations have birth and infant outcomes that are better than expected based on socioeconomic risk. Prior studies suggest that this may be related to factors such as nativity, migration patterns, health behaviors, and family or social support, although the reasons are not fully understood [15-17]. Hispanic populations are also diverse, and aggregate Hispanic estimates may hide important differences by country of origin, nativity, acculturation, and state context [15-17]. Therefore, the lower aggregate Hispanic IMR observed in this study should not be interpreted as an absence of risk or inequity, but rather as a finding that warrants more detailed study.

The observed positive correlation between jurisdiction-level IMR and MMR indicates that states with higher infant mortality also tended to have higher maternal mortality. This association supports considering maternal and infant mortality together as related population health indicators rather than as entirely separate public health problems. Potential shared determinants include poverty, insurance coverage, access to prenatal and postpartum care, availability of obstetric and neonatal services, hospital closures, rurality, chronic disease burden, and broader SDOH [4,5]. However, these mechanisms were not directly measured in the present analysis. Therefore, structural and healthcare-system explanations should be interpreted as plausible explanations supported by prior literature, not as causal findings demonstrated by this study.

Some jurisdictions differed from the overall jurisdiction-level association between IMR and MMR. For example, Wyoming had a relatively high MMR but not a similarly high IMR, illustrating that the association between these outcomes does not apply uniformly to every state. This divergence may reflect both statistical and clinical factors. Maternal mortality is a rare outcome, and jurisdiction-level MMR can be unstable in states with smaller birth populations, because a change of only one or two maternal deaths can substantially alter the calculated rate. In addition, maternal and infant mortality may be influenced by overlapping but not identical determinants. Acute maternal deaths may be more sensitive to timely access to obstetric emergency care, blood products, surgical capability, critical care, and transport distance, whereas infant mortality is influenced by a broader set of factors, including prematurity, congenital anomalies, sleep-related deaths, socioeconomic conditions, and neonatal care access. These discordant jurisdictions should therefore be viewed as hypothesis-generating, rather than as evidence against the broader population-level association between IMR and MMR.

Taken together, the main descriptive findings remained clear: higher mean mortality rates were observed in the South; Black populations experienced elevated infant and maternal mortality across jurisdictions with available data; AIAN and NHOPI populations had elevated aggregate infant and maternal mortality; and IMR and MMR were positively correlated at the jurisdiction level. These findings suggest that the observed disparities are not explained solely by isolated high- or low-rate jurisdictions, while still requiring cautious interpretation because state-level estimates for rare outcomes may be unstable when based on small numbers.

These findings contribute to the existing literature by using recent national data, aligning the primary jurisdiction-level IMR and MMR comparison across the same pooled 2020-2024 period, and expanding jurisdiction-level racial/ethnic analysis beyond White, Black, and Hispanic populations. The results do not suggest that geographic and racial disparities are new. Rather, they show that these disparities remain evident in contemporary data, and that maternal and infant mortality demonstrate similar jurisdiction-level distributions.

Overall, this analysis reinforces that maternal and infant mortality are indicators not only of clinical care, but also of broader population health and health system performance. Future studies should directly evaluate how specific state-level structural predictors, including maternity care access, insurance coverage, rurality, poverty, hospital closures, obstetric workforce availability, racial/ethnic misclassification, and measures of structural vulnerability, contribute to the observed geographic and racial/ethnic differences.

Limitations

This study has several limitations. First, the use of secondary, aggregate data limits the ability to assess individual-level risk factors and introduces the potential for ecological fallacy; therefore, findings should be interpreted as population-level associations rather than individual-level or causal relationships. Although the primary jurisdiction-level analysis used pooled 2020-2024 data for both IMR and MMR, the secondary race/ethnicity-specific analyses used pooled 2020-2023 data because the CDC Linked Birth/Infant Death Records were available only through 2023 at the time of analysis. Maternal mortality was defined using ICD-10 codes for deaths occurring during pregnancy or within 42 days postpartum, which does not capture pregnancy-related deaths occurring up to one year postpartum and may underestimate the overall burden of mortality. Pregnancy-related deaths may also be misclassified on death certificates. In addition, maternal mortality is a rare outcome, and rates based on small numerators may be unstable, particularly for smaller jurisdictions and race/ethnicity-specific groups. CDC suppression of low-count cells resulted in missing values for some jurisdictions and racial/ethnic groups, producing variable sample sizes across analyses. Finally, this study relied on unadjusted bivariable correlations and did not use multivariable modeling to control for state-level socioeconomic, policy, or healthcare infrastructure factors. State-level analyses were also unweighted, meaning that smaller and larger jurisdictions contributed equally to jurisdiction-level means and correlations. As a result, unmeasured differences in reporting practices, healthcare access, healthcare infrastructure, and population composition may have influenced the observed findings.

## Conclusions

Geographic and racial/ethnic disparities in maternal and infant mortality remain substantial in recent U.S. data, with the highest mean rates observed in Southern jurisdictions and elevated mortality affecting Black populations across jurisdictions with available data. Elevated rates were also observed among AIAN and NHOPI populations in analyses where data were available, although estimates for these groups were limited by low counts and data suppression. The positive jurisdiction-level correlation between IMR and MMR suggests that these distinct outcomes may share overlapping population-level determinants, although causal and individual-level inferences cannot be made from this ecological analysis. Future studies should examine specific structural, socioeconomic, and healthcare-system factors that may explain the parallel distribution of maternal and infant mortality across jurisdictions.

## Appendices

Appendix 1. CDC WONDER query parameters and analytic dataset structure

1. Primary overall jurisdiction-level IMR analysis, 2020-2024

Data source: CDC WONDER Underlying Cause of Death files Query date: May 3, 2026 Years included: 2020, 2021, 2022, 2023, 2024 Geography: 50 U.S. states and District of Columbia Group by: State 

Age Restriction: “Ten Year Age Groups”: <1 year (referring to Deaths occurring before age 1 year)

Infant death definition: deaths occurring before age one year Denominator source: CDC WONDER Natality data, pooled 2020-2024 Rate calculation: infant deaths / live births x 1,000

Zero values: N/A; no 0.00 IMR values were present in the primary overall jurisdiction-level analysis.

2. Primary overall jurisdiction-level MMR analysis, 2020-2024

Data source: CDC WONDER Underlying Cause of Death files Query date: May 3, 2026 Years included: 2020, 2021, 2022, 2023, 2024 Geography: 50 U.S. states and District of Columbia Group by: State Maternal death definition: ICD-10 codes A34, O00-O95, and O98-O99 Denominator source: CDC WONDER Natality data, pooled 2020-2024 Rate calculation: maternal deaths / live births x 100,000

Zero values: retained in statistical analyses 

3. Secondary sex specific IMR analysis, 2020-2024

Data source: CDC WONDER Underlying Cause of Death files Query date: May 3, 2026 Years included: 2020, 2021, 2022, 2023, 2024 Geography: 50 U.S. states and District of Columbia Group by: State and sex Sex Groupings: Male, Female

Age Restriction: “Ten Year Age Groups”: <1 year (referring to Deaths occurring before age 1 year)

Denominator source: CDC Wonder Natality data, pooled 2020-2024 Rate calculation: infant deaths / live births x 1,000

Zero values: N/A; no 0.00 values were present in the sex-specific IMR analysis.

4. Secondary race/ethnicity-specific IMR analysis, 2020-2023

Data source: CDC Linked Birth/Infant Death Records Query date: May 3, 2026 Years included: 2020, 2021, 2022, 2023 Geography: 50 U.S. states and District of Columbia Group by: State, Single Race, and Hispanic Origin

Race/ethnicity categories: White/non-Hispanic, Black/Non-Hispanic, Asian/Non-Hispanic, Native Hawaiian or Other Pacific Islander/Non-Hispanic, American Indian and Alaska Native/Non-Hispanic, All Races/Hispanic Denominator source: CDC WONDER Natality data, pooled 2020-2023, matched by state and race/ethnicity Rate calculation: infant deaths / live births x 1,000

Zero values: retained for descriptive display; excluded from state-level mean calculations and inferential analyses.

5. Secondary race/ethnicity-specific MMR analysis, 2020-2023

Data source: CDC WONDER Underlying Cause of Death files Query date: May 3, 2026 Years included: 2020, 2021, 2022, 2023 Geography: 50 U.S. states and District of Columbia Group by: State, Single Race, and Hispanic Origin 

Race/ethnicity categories: White/non-Hispanic, Black/Non-Hispanic, Asian/Non-Hispanic, Native Hawaiian or Other Pacific Islander/Non-Hispanic, American Indian and Alaska Native/Non-Hispanic, All Races/Hispanic Maternal death definition: ICD-10 codes A34, O00-O95, and O98-O99 Denominator source: CDC WONDER Natality data, pooled 2020-2023, matched by state and race/ethnicity Rate calculation: maternal deaths / live births x 100,000

Zero values: retained for descriptive display; excluded from state-level mean calculations and inferential analyses.

Handling of Suppressed, Zero, and Missing Values

CDC-suppressed cells were coded as missing and excluded from analyses requiring the suppressed value. Values reported as 0.00 were retained for descriptive reporting and graphical display when the corresponding death count was reported as zero. For the primary overall jurisdiction-level analyses, 0.00 values were retained in statistical analyses because denominators were larger and only one jurisdiction had a reported MMR value of zero. For race/ethnicity-specific jurisdiction-level analyses, 0.00 values were retained for descriptive display but excluded from state-level mean calculations and inferential analyses because they generally occurred in smaller race/ethnicity-specific strata where rates are unstable for between-state comparisons. Jurisdiction-level sample sizes, therefore, varied by analysis according to data availability after suppression and exclusion of unstable zero-value strata.

**Table 5:** Infant and maternal mortality data by race, ethnicity, and jurisdiction, 2020-2023

State / Jurisdiction	Region	Births_White	Births_Black	Births_Hispanic	Births_Asian	Births_NHOPI	Births_AIAN	Maternal Deaths_White	Maternal Deaths_Black	Maternal Deaths_Hispanic	Maternal Deaths_Asian	Maternal Deaths_NHOPI	Maternal Deaths_AIAN	Infant Deaths_White	Infant Deaths_Black	Infant Deaths_Hispanic	Infant Deaths_Asian	Infant Deaths_NHOPI	Infant Deaths_AIAN
AL	South	133,402	65,867	24,002	3,290	209	558	33	41	Suppressed	0	0	0	708	773	137	15	0	Suppressed
AK	West	18,457	985	3,119	2,079	1,241	6,960	Suppressed	Suppressed	0	Suppressed	0	Suppressed	86	Suppressed	17	11	10	81
AZ	West	122,535	17,551	136,496	10,819	873	13,695	22	Suppressed	49	Suppressed	0	13	581	181	745	53	14	106
AR	South	89,670	26,175	16,540	3,230	2,050	799	26	19	Suppressed	Suppressed	Suppressed	0	624	348	83	25	10	Suppressed
CA	West	446,139	81,027	788,623	227,015	6,290	5,173	34	29	71	19	Suppressed	Suppressed	1,339	690	3,274	601	52	37
CO	West	139,974	12,019	74,999	9,957	830	1,307	21	Suppressed	12	Suppressed	0	0	518	123	388	47	Suppressed	Suppressed
CT	Northeast	73,571	16,764	37,743	7,641	29	132	Suppressed	Suppressed	Suppressed	Suppressed	0	0	204	145	223	25	0	0
DE	South	19,728	10,980	7,605	2,329	16	60	Suppressed	Suppressed	Suppressed	Suppressed	0	0	81	114	32	Suppressed	Suppressed	0
DC	South	11,078	14,440	5,451	1,535	15	43	0	Suppressed	Suppressed	0	0	0	22	136	27	Suppressed	Suppressed	0
FL	South	361,184	183,828	286,382	26,426	620	926	53	97	52	Suppressed	0	Suppressed	1,705	1,987	1,259	108	Suppressed	Suppressed
GA	South	212,571	167,420	81,100	22,370	474	444	43	88	11	Suppressed	0	0	1,049	1,658	423	81	Suppressed	0
HI	West	11,780	1,364	10,595	15,169	5,830	109	Suppressed	0	0	Suppressed	Suppressed	0	43	10	55	61	33	0
ID	West	67,045	1,085	15,841	1,483	253	806	12	Suppressed	Suppressed	Suppressed	Suppressed	Suppressed	300	15	88	Suppressed	Suppressed	Suppressed
IL	Midwest	274,837	80,381	117,529	33,536	138	349	51	44	18	Suppressed	0	0	1,067	1,008	624	124	Suppressed	Suppressed
IN	Midwest	222,173	39,993	37,831	9,521	260	250	68	21	12	Suppressed	0	0	1,286	493	251	43	0	Suppressed
IA	Midwest	110,197	10,427	16,011	4,110	1,268	550	23	Suppressed	Suppressed	0	Suppressed	Suppressed	439	112	78	18	12	Suppressed
KS	Midwest	94,476	9,147	24,977	4,260	312	650	20	Suppressed	Suppressed	Suppressed	Suppressed	0	509	105	131	17	Suppressed	Suppressed
KY	South	161,938	20,257	16,352	4,444	349	214	50	12	0		0	0	911	216	107	13	Suppressed	0
LA	South	113,063	82,364	22,183	4,046	118	1,076	29	59	Suppressed		0	Suppressed	597	911	96	12	0	Suppressed
ME	Northeast	41,505	2,534	1,235	754	17	302	Suppressed	0	0		0	0	225	30	Suppressed	Suppressed	0	Suppressed
MD	South	109,332	80,695	55,038	17,768	125	314	23	25	Suppressed	Suppressed	0	0	424	764	277	61	Suppressed	Suppressed
MA	Northeast	150,884	27,679	59,714	22,195	76	250	24	14	Suppressed		0	0	372	190	240	60	0	Suppressed
MI	Midwest	280,593	71,184	28,742	16,371	165	1,549	45	30	Suppressed	Suppressed	0	Suppressed	1,318	934	159	89	Suppressed	Suppressed
MN	Midwest	168,286	31,133	23,345	19,472	301	3,181	19	Suppressed	Suppressed	Suppressed	0	Suppressed	594	290	96	76	Suppressed	35
MS	South	70,526	57,705	7,487	1,707	67	946	12	37	Suppressed		0	Suppressed	460	704	46	Suppressed	Suppressed	10
MO	Midwest	199,279	37,293	19,817	6,462	1,020	676	47	23	Suppressed	Suppressed	0	0	1,008	446	106	31	13	Suppressed
MT	West	35,618	256	2,598	461	47	3,863	10	0	Suppressed		0	Suppressed	147	Suppressed	18	Suppressed	0	42
NE	Midwest	64,976	6,267	18,658	3,370	169	1,090	14	Suppressed	Suppressed		0	0	328	78	107	16	0	15
NV	West	44,811	17,417	51,255	9,848	1,372	899	11	Suppressed	Suppressed	Suppressed	Suppressed	0	166	159	254	39	Suppressed	10
NH	Northeast	41,335	1,101	3,358	1,712	27	36	11	0	Suppressed		0	0	141	10	17	Suppressed	0	0
NJ	Northeast	185,588	50,714	115,517	41,276	144	165	28	37	28	Suppressed	0	0	452	441	439	97	0	Suppressed
NM	West	22,389	1,559	49,838	1,566	68	8,870	Suppressed	0	15	Suppressed	0	Suppressed	96	17	251	Suppressed	Suppressed	48
NY	Northeast	416,699	113,130	197,851	82,189	235	1,342	63	69	35	11	Suppressed	0	1,273	953	840	221	Suppressed	13
NC	South	247,662	105,766	86,418	19,146	548	5,468	59	70	17	Suppressed	0	Suppressed	1,255	1,275	463	97	Suppressed	48
ND	Midwest	28,517	2,389	2,773	864	122	2,672	Suppressed	Suppressed	0		0	Suppressed	104	17	14	Suppressed	0	20
OH	Midwest	364,264	82,682	34,707	15,642	456	370	69	51	Suppressed	Suppressed	0	0	2,000	1,089	228	72	Suppressed	Suppressed
OK	South	104,519	15,142	32,967	5,124	987	17,381	20	10	Suppressed	Suppressed	0	10	624	178	190	24	12	119
OR	West	98,623	4,048	33,648	8,345	1,361	1,471	21	0	Suppressed	Suppressed	0	Suppressed	361	35	159	28	Suppressed	Suppressed
PA	Northeast	341,439	66,248	70,527	24,294	171	474	48	27	11	Suppressed	0	0	1,487	660	419	97	Suppressed	Suppressed
RI	Northeast	22,305	3,283	11,831	1,750	14	136	Suppressed	Suppressed	Suppressed		0	0	55	20	80	Suppressed	0	0
SC	South	126,513	62,397	27,409	4,677	283	502	26	42	Suppressed		0	0	628	739	137	13	Suppressed	Suppressed
SD	Midwest	31,862	1,482	2,917	815	49	5,851	Suppressed	Suppressed	Suppressed		0	Suppressed	165	18	18	Suppressed	0	89
TN	South	209,788	59,508	41,322	7,208	303	344	80	55	12	Suppressed	Suppressed	0	1,147	653	201	25	Suppressed	0
TX	South	484,838	187,958	736,182	80,976	2,300	2,563	154	116	197	15	Suppressed	Suppressed	2,195	1,872	3,797	306	17	Suppressed
UT	West	131,024	2,429	34,674	4,698	2,148	1,346	16	Suppressed	Suppressed		0	Suppressed	616	27	184	19	17	12
VT	Northeast	18,789	484	594	547	Suppressed	53	0	0	0		0	0	67	Suppressed	Suppressed	Suppressed	0	0
VA	South	203,445	74,942	62,015	27,851	448	542	65	48	13	Suppressed	0	0	922	818	350	103	Suppressed	Suppressed
WA	West	178,707	14,989	66,615	35,437	5,044	3,619	19	Suppressed	12	Suppressed	Suppressed	Suppressed	727	114	298	111	49	36
WV	South	61,493	2,285	1,636	697	15	66	16	Suppressed	0		0	0	408	25	10	Suppressed	0	0
WI	Midwest	170,632	23,673	27,923	10,874	105	2,104	27	10	Suppressed	Suppressed	0	0	762	314	174	65	0	19
WY	West	18,819	187	3,260	257	27	683	Suppressed	0	Suppressed	Suppressed	0	0	87	Suppressed	19	Suppressed	0	Suppressed
TOTAL		7,358,878	2,050,633	3,635,250	867,613	39389*	103,229	1464	1155	681	115	15	66	32683	21911	17639	2985	311	860

**Table 6:** Overall infant and maternal mortality dataset, 2020-2024

State / Jurisdiction	Region	Births	Infant Deaths	Maternal Deaths
AL	South	289642	2089	88
AK	West	46153	308	Suppressed
AZ	West	390217	2176	111
AR	South	177346	1436	58
CA	West	2062154	8365	223
CO	West	312588	1473	50
CT	Northeast	173620	785	25
DE	South	52667	333	Suppressed
DC	South	41121	231	10
FL	South	1096163	6435	249
GA	South	624233	4116	178
HI	West	76665	374	12
ID	West	112024	554	21
IL	Midwest	644388	3649	136
IN	Midwest	397468	2671	118
IA	Midwest	181943	842	39
KS	Midwest	171531	1046	36
KY	South	261094	1604	73
LA	South	279476	2063	110
ME	Northeast	58866	330	10
MD	South	337012	1966	69
MA	Northeast	339426	1164	55
MI	Midwest	510024	3243	96
MN	Midwest	315708	1440	47
MS	South	173236	1566	65
MO	Midwest	342844	2070	95
MT	West	55606	297	16
NE	Midwest	122141	726	27
NV	West	164707	865	35
NH	Northeast	60199	219	13
NJ	Northeast	504717	1822	125
NM	West	107187	546	31
NY	Northeast	1036955	4234	225
NC	South	601696	4043	172
ND	Midwest	49019	228	11
OH	Midwest	640904	4438	157
OK	South	240236	1647	69
OR	West	197488	868	43
PA	Northeast	647817	3629	103
RI	Northeast	50648	216	13
SC	South	287206	2025	87
SD	Midwest	56182	392	12
TN	South	409525	2617	171
TX	South	1910298	10694	571
UT	West	229865	1169	28
VT	Northeast	25921	103	0
VA	South	472907	2791	153
WA	West	414380	1888	64
WV	South	85078	586	21
WI	Midwest	301864	1730	53
WY	West	30493	148	11
Total		18170648	100250	4201

**Table 7:** Gender jurisdiction-level Infant mortality dataset, 2020-2024

State / Jurisdiction	Births_Male	Births_Female	Infant Deaths _ Male	Infant Deaths_ Female
AL	147558	142084	1179	910
AK	23673	22480	187	121
AZ	199466	190751	1174	1002
AR	90585	86761	770	666
CA	1054389	1007765	4607	3758
CO	159840	152748	799	674
CT	88943	84677	460	325
DE	27010	25657	181	152
DC	20954	20167	118	113
FL	560611	535552	3520	2915
GA	318962	305271	2268	1848
HI	39423	37242	212	162
ID	57428	54596	295	259
IL	329294	315094	2015	1634
IN	203459	194009	1516	1155
IA	92946	88997	480	362
KS	87632	83899	576	470
KY	133348	127746	853	751
LA	142820	136656	1119	944
ME	30214	28652	175	155
MD	171434	165578	1045	921
MA	173801	165625	650	514
MI	261018	249006	1785	1458
MN	161601	154107	792	648
MS	88332	84904	873	693
MO	175471	167373	1127	943
MT	28560	27046	165	132
NE	62110	60031	398	328
NV	84216	80491	483	382
NH	30835	29364	121	98
NJ	258320	246397	1023	799
NM	54779	52408	309	237
NY	530121	506834	2325	1909
NC	307975	293721	2244	1799
ND	25208	23811	138	90
OH	327742	313162	2426	2012
OK	122815	117421	920	727
OR	100946	96542	483	385
PA	331729	316088	1983	1646
RI	25738	24910	125	91
SC	146563	140643	1097	928
SD	28807	27375	202	190
TN	208860	200665	1473	1144
TX	976243	934055	5874	4820
UT	117469	112396	644	525
VT	13311	12610	57	46
VA	241858	231049	1506	1285
WA	211355	203025	1047	841
WV	43455	41623	336	250
WI	154913	146951	922	808
WY	15577	14916	70	78
Total	9289717	8880931	55147	45103
